# Immunosuppressant treatment reduces cocaine-induced behavioral sensitization in mice

**DOI:** 10.3389/fphar.2026.1757023

**Published:** 2026-02-10

**Authors:** Gabriella Luciana de Oliveira, Maria Carolina Machado da Silva, Giovanni Freitas Gomes, Roberta dos Santos Ribeiro, Gabriela Reis Cussat, Rúbia Aparecida Fernandes, Jorge Lucas Nascimento Souza, Heliana de Barros Fernandes, Aline Silva de Miranda, Victor Rodrigues Santos, Lílian Lacerda Bueno, Luciene Bruno Vieira, Fabrício Araújo Moreira, Antônio Carlos Pinheiro de Oliveira

**Affiliations:** 1 Department of Pharmacology, Institute of Biological Sciences (ICB), Universidade Federal de Minas Gerais (UFMG), Belo Horizonte, Minas Gerais, Brazil; 2 Department of Morphology, Institute of Biological Sciences (ICB), Universidade Federal de Minas Gerais (UFMG), Belo Horizonte, Minas Gerais, Brazil; 3 Department of Parasitology, Institute of Biological Sciences (ICB), Universidade Federal de Minas Gerais (UFMG), Belo Horizonte, Minas Gerais, Brazil

**Keywords:** calcineurin, cocaine, FK506, neuroinflammation, plasticity, tacrolimus

## Abstract

**Background:**

Neuroinflammation plays fundamental, though still not fully understood, roles in the pathophysiology of substance use disorders, including cocaine addiction. Chronic cocaine exposure promotes neuroinflammatory signaling and synaptic alterations in brain regions involved in reward and memory, such as the striatum and hippocampus. Among the intracellular pathways regulating these processes, calcineurin, a calcium calmodulin-dependent phosphatase, has been implicated in synaptic plasticity, neuroinflammation, and psychiatric disorders. FK506 (tacrolimus), a calcineurin inhibitor and immunosuppressant drug used in the clinics, modulates neurotransmitter release, neurotrophic factor production, and microglial activity. However, its role in cocaine-induced neuroinflammatory and behavioral alterations remains poorly defined. In this context, we sought to evaluate whether FK506 alters the development of cocaine-induced behavioral, molecular, inflammatory, and structural alterations in C57Bl/6 male mice.

**Methods:**

Male C57Bl/6 mice (9–11 weeks) received FK506 (5 mg/kg, s.c.) or saline and were submitted to locomotor sensitization induced by repeated cocaine administration (15 mg/kg, i.p.). The hippocampus and striatum were collected for quantification of GDNF, TNF, IL-10, and IL-6 by ELISA, and for qPCR analyses of neuronal activity and plasticity related genes (PSD95, FosB, CREB, and ARC). Dendritic spine density was evaluated in the dentate gyrus and nucleus accumbens.

**Results:**

In male mice, FK506 attenuated cocaine-induced locomotor sensitization from the fourth day. The drug decreased hippocampal levels of GDNF, TNF-α, and IL-10 relative to the cocaine group, albeit no corresponding reductions were detected in the striatum. Consistent with this, FK506 neither altered plasticity- and activity-related gene expression nor reversed cocaine- induced dendritic spine loss.

**Conclusion:**

Together, these findings indicate that the immunosuppressant partially modulates cocaine’s effects, primarily by reducing the behavior sensitization and influencing specific neuroinflammatory and neurotrophic responses. Even without reversing structural or transcriptional alterations, the results suggest that immunomodulatory interventions may influence specific neurobiological adaptations to cocaine and warrant further investigation as potential therapeutic strategies.

## 1 Introduction

Substance use disorder (SUD) is a chronic and relapsing condition characterized by compulsive drug seeking and consumption despite adverse consequences, reflecting a dysregulation of neural circuits that govern reward, stress, and self-control (Sinha, 2008; American Psychiatric Association, 2014). SUD is recognized as a neurobiological disease involving persistent functional and structural alterations associated with maladaptive neuroplasticity in limbic and cortical regions (Corominas et al., 2007; Volkow et al., 2019). Among psychostimulants, cocaine stands out for its high potential for abuse and relapse, representing a major global public health concern (United Nations Office on Drugs and Crime, 2025).

Cocaine exerts its primary pharmacological effects by inhibiting monoamine transporters, particularly the dopamine transporter (DAT), leading to elevated extracellular dopamine levels and enhanced activation of postsynaptic receptors (Fleckenstein et al., 2007; Koob and Volkow, 2016). This disruption of dopaminergic signaling within the mesocorticolimbic pathway encompassing the ventral tegmental area (VTA), nucleus accumbens (NAc), and prefrontal cortex (PFC) underlies the reinforcing and motivational properties of the drug (Robinson and Berridge, 1993; Koob and Volkow, 2010). Repeated cocaine exposure induces behavioral sensitization, manifested as a progressive amplification of locomotor activity, which reflects enduring synaptic and molecular adaptations associated with relapse vulnerability (Sorg and Ulibarri, 1995; Robinson and Kolb, 1999). This experimental model is widely employed because it recapitulates key neurobiological features of SUD, allowing the investigation of neurobiological mechanisms underlying neuroplasticity and neuroinflammation (Fleckenstein et al., 2007; Gulley and Stanis, 2010; Schierberl et al., 2011; Koob and Volkow, 2016). In parallel, associative reward learning represents a core component of cocaine use disorder, as environmental cues paired with drug exposure acquire strong motivational salience and contribute to craving and relapse (Saunders and Robinson, 2011). The conditioned place preference (CPP) paradigm is widely used to capture this process, as models the formation and retrieval of cocaine-associated contextual memories that depend on coordinated activity within the NAc, hippocampus, and PFC (Tzschentke, 2007). Thus, the combined use of locomotor sensitization and CPP provides a more comprehensive assessment of both psychomotor and reward-learning dimensions of cocaine-induced neuroadaptations.

From a neuroanatomical standpoint, cocaine-induced neuroadaptations are distributed across multiple nodes of the mesocorticolimbic circuitry, including the striatum, particularly the NAc, the medial prefrontal cortex (mPFC), and the hippocampus, regions critically involved in reward processing, executive control, and associative learning (Hyman et al., 2006). In these areas, chronic cocaine exposure results in extensive synaptic remodeling, changes in dendritic spine density, and modifications in interneuron populations, and impaired neurogenesis, indicating widespread structural and functional reorganization (Mash et al., 2007; Smith et al., 2013; Ray et al., 2015; Ladrón de Guevara-Miranda et al., 2017).

Beyond synaptic alterations, accumulating evidence indicates that neuroinflammation plays a central role in the pathophysiology of cocaine use disorder. Chronic cocaine exposure induces microglial activation, astrocytic reactivity, and sustained neuroimmune signaling, which contribute to synaptic dysfunction and maladaptive plasticity (Lewitus et al., 2016; Wang et al., 2022; Mongrédien et al., 2025; Saint-Jour et al., 2025). Indeed, we have demonstrated that microglia depletion reduced locomotor sensitization induced by cocaine (da Silva et al., 2021).

In humans, various studies have demonstrated altered levels of cytokines in the plasma of cocaine users, such as TNF, IL-1beta, IL-2, IL-6, IL-10, IFN-gamma, CXCL12, CX3CL1 and others (Fiala et al., 1996; Araos et al., 2015; Moreira et al., 2016; Maza-Quiroga et al., 2017; Zaparte et al., 2019; Stamatovich et al., 2021). Interestingly, it has been shown that some cytokines correlated with the severity of cocaine symptoms, indicating a possible association with clinical manifestations of this drug use (Araos et al., 2015; Stamatovich et al., 2021). Cytokines also correlate with the severity of withdrawal symptoms (Zaparte et al., 2019).

In mice, chronic administration of cocaine increases not only microglial activation and altered the expression of pro-inflammatory cytokines within the striatum and hippocampus (Ezeomah et al., 2022; Cheng et al., 2023), but also the production of these inflammatory mediators by splenocytes and peripheral macrophages (Wang et al., 1994). These mediators interfere with glutamatergic transmission, impair long-term synaptic plasticity, and exacerbate neurotoxicity, thereby reinforcing behavioral inflexibility and relapse vulnerability (Roodsari et al., 2022). Conversely, anti-inflammatory mediators, particularly IL-10, can counteract these effects by reducing TNF release and modulating the inflammatory cascade (Armstrong et al., 1996; Yan et al., 2014).

Cocaine also modulates different intracellular signalling pathways, which, in turn, could contribute to the neuroadaptations involved in the SUD. Among these intracellular pathways, calcineurin, a calcium/calmodulin-dependent serine/threonine phosphatase could contribute to this condition. In other conditions not associated with SUD, it has been shown that this phosphatase plays a central role in the regulation of transcription factors and synaptic proteins essential for long-term potentiation (LTP), long-term depression (LTD), and memory processing (Mulkey et al., 1994; Aramburu et al., 2004; Xia and Storm, 2005). In rats, 5-day administration of cocaine slightly reduces the expression of calcineurin in the NAc (Hu et al., 2005). In addition, it has been shown that activation of calcineurin in the lateral amygdala reduced cocaine cue–induced reinstatement and weakened thalamo–amygdala synaptic strength by modulating memory reconsolidation and extinction (Rich et al., 2020). It has also been shown that cocaine reduces calcineurin signaling in the NAc, leading to inhibition of myocyte enhancer factor 2 activity and thereby promoting dendritic spine formation and cocaine-induced behavioral plasticity (Pulipparacharuvil et al., 2008). This phosphatase is an important downstream effector of D2 receptor signaling in the NAc and contributes to cocaine-induced modulation of neuronal excitability (Perez et al., 2011). Finally, it has been shown that calcineurin signaling regulates cocaine-induced expression of Npas4, a transcription factor that is induced by neuronal activity, in the nucleus accumbens, thereby influencing synaptic adaptations and behavioral responses to cocaine (Lissek et al., 2021).

Pharmacological inhibition of calcineurin by compounds such as tacrolimus (FK506) is known to modulate cytokine expression in the central nervous system (CNS) (Van der Perren et al., 2015; Shah et al., 2019). In parallel, inhibition of this phosphatase enhances NMDA receptor activity and extends the duration of LTP, suggesting a facilitatory effect on synaptic plasticity (Hoeffer et al., 2007; Zhou et al., 2022). These actions have been reported in several brain regions, including the hippocampus and VTA, in models of neurodegeneration and psychostimulant exposure (Biała et al., 2005; Addy et al., 2007; Gomes et al., 2025).

Therefore, the present study investigated the effects of FK506 on cocaine-induced behavioral and molecular alterations, employing the locomotor sensitization and CPP models in addition to analyses of cytokines and plasticity-related markers in the striatum and hippocampus. FK506 is a FDA-approved immunosuppressant widely used to prevent transplant rejection and to treat autoimmune diseases. Considering its established actions on intracellular signaling, neuroinflammation, and synaptic plasticity, we hypothesized that FK506 could be repurposed to modulate neurobiological and behavioral outcomes associated with chronic cocaine exposure. By probing these interactions, our study aims to shed light on the potential of calcineurin modulation as a therapeutic strategy for substance use disorders.

## 2 Materials and methods

### 2.1 Animals

Male and female C57Bl/6 mice, obtained from the Federal University of Minas Gerais, were used in this study. The animals, aged between 9 and 12 weeks, had *ad libitum* access to water and food and were maintained on a 12-h light/dark cycle at an ambient temperature of approximately 24 °C. Female mice were randomly assigned to experimental groups, and the estrous cycle was not controlled during treatments or tissue collection (Prendergast et al., 2014). All experiments were conducted in accordance with the ethical principles for animal experimentation adopted by the Institutional Animal Care and Use Committee (CEUA) and were approved under protocol number 261/2021.

### 2.2 Drugs

The animals were treated with FK506 (Tacrolimus; LC Laboratory, United States), which was diluted in a solution of 1% DMSO, 5% Tween 80, and sterile saline, at a dose of 5 mg/kg, administered via subcutaneous (s.c.) injection 80 min before each behavioral test. The dose of FK506 was selected based on previous studies, including prior work from our group, in which FK506 was evaluated as a pharmacological repurposing candidate and shown to produce consistent behavioral and neurobiological effects without inducing nonspecific toxicity or locomotor impairment (Udina et al., 2003; Gold et al., 2004; Fields et al., 2016; He et al., 2021; Gomes et al., 2025). Cocaine (Merck and Co., Inc.), diluted in saline solution at a dose of 15 mg/kg, was administered via intraperitoneal (i.p.) injection immediately before each behavioral test.

### 2.3 Behavioral sensitization

**FIGURE 1:**
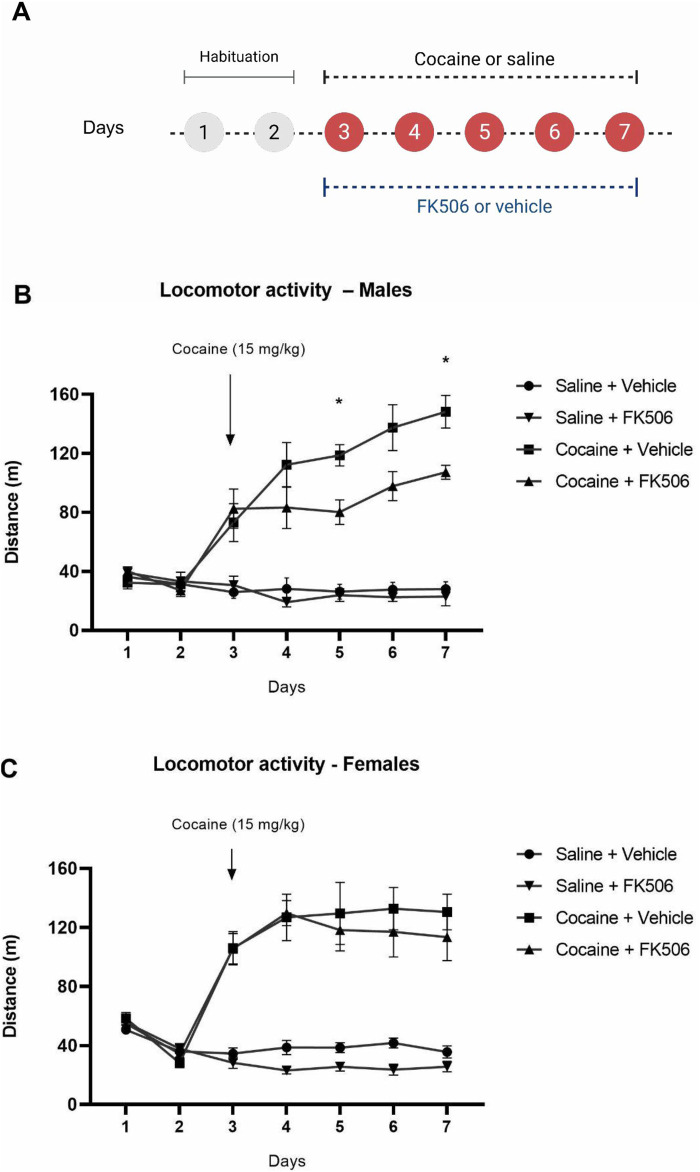
Locomotor activity during behavioral sensitization after FK506 treatment in male and female mice. **(A)** Experimental timeline showing the habituation phase (days 1–2) and treatment phase (days 3–7) as described in Section 2.3. **(B)** Locomotor activity of male mice treated with FK506 + cocaine (n = 9), vehicle + cocaine (n = 9), FK506 + saline (n = 8), and vehicle + saline (n = 8) across the 7-day behavioral sensitization protocol. Data were analyzed using two-way repeated-measures ANOVA followed by Tukey’s *post hoc* test. *p < 0.05 Cocaine + Vehicle vs. Cocaine + FK506 on days 5 and 7. **(C)** Locomotor activity of female mice treated with FK506 + cocaine (n = 10), vehicle + cocaine (n = 9), FK506 + saline (n = 8), and vehicle + saline (n = 8) over the same 7-day sensitization procedure. Data were analyzed using two-way repeated-measures ANOVA followed by Tukey’s *post hoc* test.

The animals were subjected to an open field test to evaluate locomotor activity, where they were placed individually in the center of the arena and the total distance traveled was measured over 30 min for 7 days. The open field apparatus consisted of a circular arena approximately 120 cm in diameter, surrounded by a circular wall 45 cm in height. On days 1 and 2, the animals were habituated to the arena. From days 3 to 7, the animals were treated with FK506 or vehicle subcutaneously at a dose of 5 mg/kg (Figure 1A). After 80 min, the animals were administered cocaine or saline intraperitoneally at a dose of 15 mg/kg and were immediately placed in the apparatus (n = 9 animals per group). The test was recorded and analyzed using ANY-maze software (version 6.0).

### 2.4 Conditioned-place preference

For the conditioned place preference (CPP) test, we used an acrylic chamber divided into three compartments, each lateral compartment displaying distinct wall and floor patterns. The apparatus consisted of a central neutral compartment (9.5 cm × 5 cm × 12 cm) connecting two lateral conditioning compartments of equal dimensions (15 cm × 12 cm × 12 cm). The lateral compartments were differentiated by distinct visual and tactile cues: one compartment featured horizontally oriented black-and-white striped walls and a floor with linear textured grooves, whereas the opposite compartment presented vertically oriented striped walls and a perforated acrylic floor. The lateral compartments were connected by a neutral central corridor with removable doors, allowing either confinement to a specific compartment or free access to the entire apparatus. The CPP protocol consisted of three phases: pre-test, conditioning, and test. In the pre-test (day 1), mice were individually placed in the central corridor and allowed to freely explore all compartments for 15 min; sessions were recorded to determine baseline preference. Conditioning occurred over 6 days. Animals received FK506 or vehicle (5 mg/kg, s.c.) once daily, followed 80 min later by cocaine or saline (15 mg/kg, i.p.) on alternating days (n = 15 animals per group). Thus, on days 2, 4, and 6, mice received cocaine and were immediately confined to one compartment for 30 min; on days 3, 5, and 7, they received saline and were confined to the opposite compartment. Control animals received saline throughout. On the test day (day 8), mice were again placed in the central corridor and allowed to freely explore the apparatus for 15 min (Supplementary Figure S1A). An unbiased CPP paradigm was employed, as baseline preference during the pre-test did not influence compartment assignment. CPP performance was analyzed using ANY-maze software, and the CPP score was calculated as a percentage according to the formula: [(A2 − A1) × 100]/(A2 + A1), where A2 represents the time spent in the drug-paired compartment during the test session and A1 represents the time spent in the saline-paired compartment.

### 2.5 Evaluation of neurotrophic factors and cytokine levels

Following the locomotor sensitization, transcardiac perfusion was performed for subsequent brain tissue collection. For this, the animals were anesthetized with xylazine (8 mg/kg, i.p.) and ketamine (80 mg/kg, i.p.) and perfused with phosphate-buffered saline (PBS, pH 7.4). The animals were then decapitated, and the brain was removed, dissected to collect the hippocampus and striatum, and stored at −80 °C. Molecular analyses were performed exclusively in male mice. The dissected samples were homogenized in a cytokine extraction buffer [2 mM Tris-HCl, pH 8.0; 337 mM NaCl; 1% NP-40; 10% glycerol; 0.1 mM phenylmethylsulfonyl fluoride (PMSF); 1 μM pepstatin A; 10 mM EDTA; 10 μM E-64; and 0.5 mM sodium orthovanadate, diluted in distilled water]. The samples were then centrifuged at 4 °C at 10,000 rpm for 20 min, aliquoted, and stored at −20 °C. Enzyme-linked immunosorbent assay (ELISA) was performed to quantify cytokines [interleukin-6 (IL-6), IL-10, and tumor necrosis factor (TNF), fractalkine (CX3CL1)] and neurotrophic factors [brain-derived neurotrophic factor (BDNF), and nerve growth factor (NGF) and glial cell line-derived neurotrophic factor (GDNF)] (n = 8 animals per group). Levels were measured using commercial kits from the R&D Systems (DuoSet, Minneapolis, MN) according to the manufacturer’s instructions. The results were obtained using a spectrophotometer and expressed as pg/mL.

### 2.6 Dendritic spine analysis

The animals were subjected to transcardiac perfusion for brain tissue fixation. After anesthesia, perfusion was initiated with 0.1 M phosphate-buffered saline (PBS) to remove circulating blood, followed by brains extraction and post-fixation in 4% paraformaldehyde (PFA) overnight at 4 °C. The brains were then transferred to 0.1 M PBS and stored at 4 °C until sectioning. Coronal sections (150 µm thick) were obtained using a vibratome (Leica VT1000S). Neuronal impregnation and staining were performed with the sliceGolgi Kit (Bioenno Tech, LLC, Cat. No. 003760), according to the manufacturer’s instructions. The sections were mounted on gelatin-coated microscope slides and, after drying, followed by dehydration in absolute ethanol and clearing in xylene. Finally, the slides were coverslipped using Entellan Novo mounting medium (Sigma-Aldrich, Lot: HX32559461). Morphological analysis of dendritic spines was conducted in the nucleus accumbens and dentate gyrus regions using an optical microscope (Zeiss Axiovert 5). The images were processed in ImageJ software (NIH, United States). For each selected dendrites, a line of known length (µm) was manually traced, and all visible spines along this segment were counted. Spines density was calculated as the total number of spines divided by dendritic length and expressed as spines/µm (n = 6 animals per group).

### 2.7 RNA extraction and qPCR

Hippocampal and striatal samples were processed for gene expression analysis (n = 6 animals per group). Total RNA was isolated using NucleoSpin® RNA Plus Kit (Macherey-Nagel, Düren, Germany) according to the manufacturer’s instructions using 30 mg of tissue. RNA purity and concentration were determined in NanoDrop 2000 (Thermo Fisher Scientific Inc., United States). Samples presenting 260/280 nm and 260/230 nm absorbance ratios within the range of 1.8–2.1 were considered for subsequent cDNA synthesis. Thus, 2 µg of RNA from each sample was reverse-transcribed into cDNA using High-Capacity cDNA Reverse Transcriptase Kit (Applied Biosystems Inc., Foster City, CA) in a total volume of 20 µL according to the manufacturer’s instructions. The cDNA was diluted 1:10 for subsequent qPCR. The qPCR reactions were carried out using GoTaq® qPCR Master Mix (Promega, Madison, WI, United States) following the manufacturer’s instructions. Relative expression levels were calculated using the 2^−ΔCt method, and data are expressed as normalized values relative to the saline + vehicle control group.

### 2.8 Primer design and validation

Primers targeting genes related to neuronal activity and synaptic plasticity (PSD95, FosB, CREB, and ARC) were used, with GAPDH serving as a reference gene. GAPDH sequences were obtained in (Souza et al., 2024) while the other primer sequences (forward and reverse) were designed using mouse nucleotide sequences retrieved from NCBI, according to the accession numbers listed in Table 1. Primers were generated with Primer3 (v4.1.0) using the following parameters: length 18–22 bp, melting temperature 59 °C–61 °C, GC content 40%–60%, and amplicon size 90–200 bp. Primer quality was evaluated with RNAfold and Autodimer to exclude secondary structures and primer-dimers. Specificity was verified by aligning the predicted amplicons using BLAST. Serial dilutions of the samples were prepared for each primer pair to determine amplification efficiency (E%) and the coefficient of determination (R^2^), which were obtained from the resulting standard curves. Linear regression was used to calculate the slope, from which efficiency was derived using the formula E% = 10^(−1/slope)^ × 100. Primer pairs with efficiencies between 90% and 115% were considered acceptable. The specificity of each amplified product was verified by confirming the expected amplicon size on a 2% agarose gel. All primer sequences are provided in Table 1.

**TABLE 1:** Primer sequences and qPCR validation parameters.

Primer	Sequence	Amplicon length (Pb)	Slope	E (%)	R^2^	Tm (°C)	NCBI accession number
PSD95 (DLG4)	F: TCCAGTCTGTGCGAGAGGTA R: GGACGGATGAAGATGGCGAT	118 pb	−3,73	90,60	0.963	87,28	NM_001109752.1
FosB	F: ACCCACCCTCATCTCTTCCA R: GTAGCTGGTTCCTGGCATGT	97 pb	−3,1	110,17	0.976	85,47	NM_001347586.1
CrebBP	F: TGCCACATCACAGACTGGAC R: GCAGCCCCAAGAGATCCATT	150 pb	−3,15	107,71	0.895	83,5	NM_001025432.1
Arc	F: GATCTTTCCTGCTGTGCCCT R: AGGTTTCAGCTGGGCAATCA	121 pb	−3,38	97,63	0.868	87,08	NM_001276684.1

Forward (F) and reverse (R) primer sequences, expected amplicon size, melting temperature (Tm), amplification efficiency (E%), slope, R^2^, and NCBI, accession numbers for each target gene used in the study.

### 2.9 Statistical analyses

For statistical analysis, GraphPad Prism 8.0 software (GraphPad, CA, United States) was utilized. Data normality was assessed using the Kolmogorov-Smirnov test. Depending on the data, one-way ANOVA, two-way ANOVA, or two-way repeated-measures ANOVA was conducted, followed by Tukey’s *post hoc* test. Results are presented as mean ± SEM, and differences between groups were considered statistically significant when p < 0.05.

## 3 Results

### 3.1 FK506 significantly attenuates cocaine-induced locomotor sensitization in males, but not females

We first assessed the effects of FK506 using locomotor sensitization (Figure 1A), a well-established paradigm for modeling cocaine-induced neuroadaptations and predicting vulnerability to substance use disorders. In males, two-way repeated-measures ANOVA revealed significant effects of treatment (F (3,27) = 48.24, p < 0.0001), time (F (6,162) = 26.38, p < 0.0001), and a treatment × time interaction (F (18,162) = 15.57, p < 0.0001). Cocaine increased locomotor activity from day 3, and FK506 significantly reduced this psychostimulant-induced hyperlocomotion on days 5 (p = 0.0182) and 7 (p = 0.0365). Despite this attenuation, locomotor sensitization was not fully abolished, remaining significantly different in the Cocaine + FK506 group compared to its respective control (p < 0.05) (Figure 1B). FK506 alone did not affect locomotion relative to controls. In females, two-way repeated-measures ANOVA also showed significant effects of treatment (F (3,29) = 30.02, p < 0.0001), time (F (6,174) = 19.10, p < 0.0001), and a treatment × time interaction (F (18,174) = 11.30, p < 0.0001). Cocaine similarly induced locomotor sensitization beginning on day 3; however, FK506 did not attenuate this effect at any time point (Figure 1C).

### 3.2 FK506 did not reverse cocaine-induced conditioned place preference in male or female mice

To further investigate the effects of FK506 in the altered behavior induced by cocaine, we employed the CPP paradigm (Supplementary Figure S1A), a complementary model that assesses distinct aspects of drug-induced associative learning and reward. In males, cocaine produced a robust conditioned place preference relative to saline controls (F (1,52) = 34.16; p < 0.0001; *post hoc* p < 0.0001), and cocaine-induced conditioned place preference was preserved in FK506-pretreated animals, with cocaine-treated mice differing from saline controls (p < 0.001). However, no difference was observed between cocaine-treated animals receiving vehicle or FK506 (p = 0.6675) (Supplementary Figure S1B). In females, cocaine similarly induced strong place preference (F (1,52) = 91.62; p < 0.0001; *post hoc* p < 0.0001), which was also maintained in FK506-pretreated animals, with cocaine-treated mice differing from saline controls (p < 0.0001). In addition, no difference was observed between cocaine-treated animals receiving vehicle or FK506 (p = 0.8801) (Supplementary Figure S1C).

### 3.3 FK506 reduced TNF and IL-10 levels in mice treated with cocaine in the hippocampus

**FIGURE 2:**
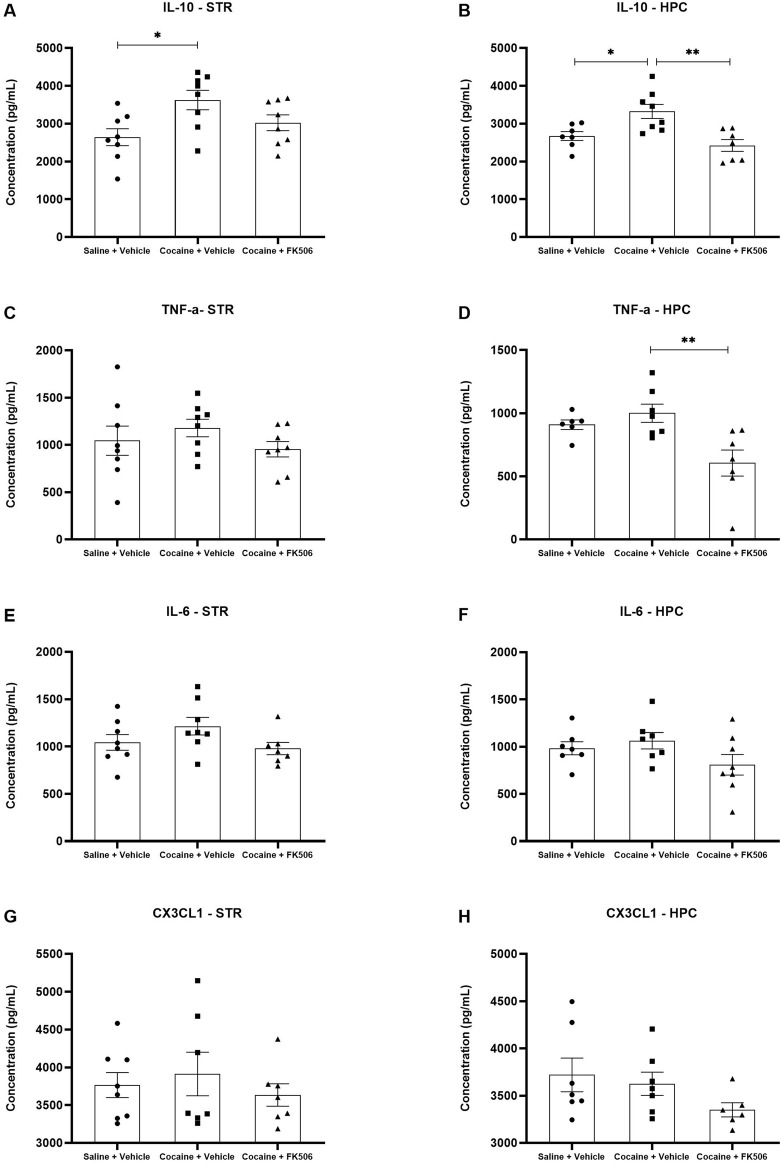
Levels of cytokines in the striatum (STR) and hippocampus (HPC). Quantification of IL-10 **(A,B)**, TNF **(C,D)**, IL-6 **(E,F)** and CX3CL1 **(G,H)** levels in the striatum **(A,C,E,G)** and hippocampus **(B,D,F,H)** of male mice following treatment with FK506 and cocaine. Data are expressed as mean ± SEM (n = 8 per group). *p < 0.05 compared with the cocaine group, as determined by one-way ANOVA followed by Tukey’s *post hoc* test.

Since FK506 did not modify cocaine-induced conditioned place preference, we next analyzed brain samples from the locomotor sensitization experiment to investigate whether the drug could influence neuroinflammatory mediators associated with cocaine exposure. ELISAs were performed to quantify TNF, IL-10, IL-6 and CX3CL1 in the hippocampus and striatum. In the hippocampus, cocaine increased IL-10 levels compared with saline controls, and FK506 significantly attenuated this cocaine-induced elevation (F (2,19) = 8.754; p < 0.05, Figure 2B). Although cocaine did not increase TNF at this time point, FK506 reduced the levels of this cytokine in cocaine-treated animals relative to the cocaine group (Figure 2D). No differences were observed across groups for IL-6 and CX3CL1 (Figures 2F,H). In the striatum, cytokine levels remained unchanged among all experimental groups, indicating that the effects of FK506 on the cytokine’s levels were region-specific (Figures 2A,C,E,G).

### 3.4 Cocaine-induced reduction in dendritic spine density is not prevented by FK-506

**FIGURE 3:**
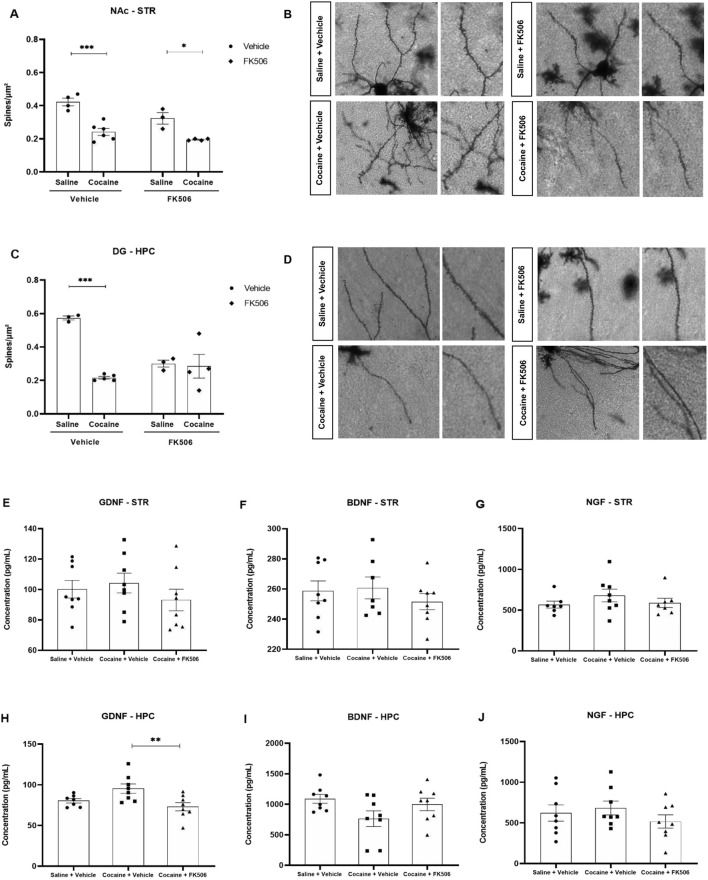
Dendritic spine density and neurotrophic factor levels in the STR and HPC. Golgi-Cox staining in the striatum (STR), specifically in the nucleus accumbens (NAc) **(A)**, and in the hippocampus (HPC), specifically in the dentate gyrus (DG) **(C)** of male mice. Representative dendritic segments from each experimental group are shown (**(B)** NAc; **(D)** DG). Spine density was quantified in mice treated with cocaine + FK506 (n = 6), cocaine + vehicle (n = 6), FK506 + saline (n = 4) and saline + vehicle (n = 4). Levels of the neurotrophic factors GDNF, BDNF, and NGF were quantified in the striatum **(E–G)** and hippocampus **(H–J)** of male mice subjected to behavioral sensitization. Animals were treated with saline + vehicle (n = 8), cocaine + vehicle (n = 8), or cocaine + FK506 (n = 8). Data were analyzed using two-way ANOVA followed by Tukey’s *post hoc* test. *p < 0.05; ***p < 0.001.

Structural plasticity is a key substrate of cocaine-induced neuroadaptations, and alterations in dendritic spine density are consistently reported in addiction-related circuits. Because FK506 reduced cocaine-induced behavioral sensitization, we next examined whether this immunosuppressant could influence the structural remodeling triggered by cocaine. Thus, dendritic spine density was quantified in the NAc of the striatum and in the dentate gyrus (DG) of the hippocampus using Golgi-Cox staining, a method that enables the visualization of neuronal morphology. In the striatum, animals treated with cocaine, receiving or not FK506, exhibited markedly lower spine density compared with saline-treated controls (F (1, 13) = 46.15, p < 0.05, Figures 3A,B). FK506 did not alter the reduction of dendritic spines induced by cocaine. In the hippocampus, cocaine administration significantly reduced dendritic spine density compared with saline-treated controls (p < 0.05, Figures 3C,D). Interestingly, FK506 alone also decreased spine density relative to saline in this brain region, indicating that the drug, *per se*, exerts a spine-reducing effect. In addition, when cocaine was administered to FK506-treated mice, no further reduction was observed, suggesting that the immunosuppressant had already driven spine density to a minimal level beyond which cocaine produced no additional effect.

### 3.5 FK506 reduced GDNF levels in mice treated with cocaine in the hippocampus

Neurotrophic factors, which are essential regulators of neuronal growth, survival, and synaptic adaptation, have been implicated in the neurobiological changes associated with cocaine-induced sensitization. Therefore, we quantified hippocampal and striatal levels of GDNF, BDNF, and NGF by ELISA in animals subjected to behavioral sensitization. In the hippocampus, FK506-treated cocaine animals displayed a significant reduction in GDNF levels compared with the cocaine + vehicle group (F (2,20) = 5.799; p = 0.0150), whereas no significant differences were detected for BDNF (p = 0.0955) or NGF (p = 0.4303) (Figures 3H–J). In the striatum, levels of GDNF (p = 0.4802), BDNF (p = 0.8080), and NGF (p = 0.5622) did not differ significantly among experimental groups (Figures 3E–G) at the time point that we evaluated the brain tissues.

### 3.6 FK506 treatment does not modulate the expression of synaptic plasticity markers

Considering that repeated cocaine exposure induces transcriptional adaptations associated with synaptic remodeling, we next examined whether FK506 could alter molecular markers of neuronal plasticity and synaptic structural remodeling. Following five consecutive days of cocaine exposure and FK506 administration, qPCR was performed to assess the expression of PSD-95, CREB, Arc, and FosB. At the time point that we evaluated the brain tissues, neither cocaine alone nor the FK506 + cocaine combination produced measurable transcriptional alterations in these genes in the striatum or hippocampus, and FK506 also failed to modulate their expression (Supplementary Figures S2A–H).

## 4 Discussion

This study investigated the effects of an immunosuppressant on the neuropathological effects induced by cocaine in mice. Here we show that i. cocaine induced locomotor sensitization, an effect that was attenuated by FK506 in males but not in females; ii. cocaine induced conditioned place preference, an effect that was not reduced by FK506; iii. FK506 also failed to alter the reduction in synaptic plasticity induced by cocaine; iv. FK506 reduced hippocampal concentrations of GDNF, TNF, and IL-10 in cocaine-treated animals.

Behavioral sensitization is a well-established paradigm for assessing long-lasting neuroadaptations resulting from repeated psychostimulant exposure. The progressive enhancement of cocaine-induced locomotion reflects enduring alterations in dopaminergic signaling and synaptic plasticity within reward-related circuits (Robinson and Berridge, 1993; Vanderschuren and Everitt, 2004). In the present study, FK506 effectively attenuated cocaine-induced hyperlocomotion in males. This could suggest that calcineurin contributes to the neurobiological processes underlying behavioral sensitization. Indeed, comparable findings have been reported for other psychostimulants, including amphetamine and nicotine, further supporting a role for calcineurin-dependent signaling in drug-induced plasticity (Tsukamoto et al., 2001; Addy et al., 2007). In contrast, these results differ from those reported by Addy et al. (2007), in which inhibition of calcineurin with cyclosporine potentiated early locomotor responses to cocaine and enhanced acute cocaine-induced activity following repeated pretreatment. This apparent discrepancy may reflect important methodological and pharmacological differences between studies, including the use of distinct calcineurin inhibitors, dosing regimens, timing of behavioral assessment, and duration of drug exposure. Notably, the authors demonstrated that calcineurin inhibition mimicked cocaine-induced phosphorylation of synapsin I, suggesting a predominant presynaptic mechanism regulating neurotransmitter release (Addy et al., 2008). In contrast, the attenuation of hyperlocomotion observed with FK506 in the present study may involve additional or downstream effects on synaptic remodeling, intracellular signaling, or neurotrophic pathways, potentially shifting the balance toward reduced behavioral output despite cocaine exposure.

Interestingly, FK506 did not attenuate locomotor sensitization in females, highlighting an important sex-dependent variability in behavioral and neurobiological responses. This includes not only the differences in the sensitivity to psychostimulants (Yamamoto et al., 2013), but also sex dependent responses to immunomodulatory treatments (Dunn et al., 2024). In ovariectomized female rats, acute estradiol administration rapidly reduced hippocampal calcineurin and protein phosphatase 1 activity, without long-lasting changes in expression, indicating a fast, non-genomic mechanism. This reduction in calcineurin activity was associated with enhanced CA3-CA1 synaptic transmission, blockade of long-term depression, and facilitation of long-term potentiation. These effects were not prevented by estrogen receptor antagonism, suggesting receptor-independent modulation of Ca^2+^-dependent phosphatase signaling (Sharrow et al., 2002). Together, these findings suggest that estradiol can rapidly shift synaptic plasticity by suppressing calcineurin activity, providing a mechanistic basis for sex hormone–dependent modulation of neural plasticity and cognitive processes. Thus, the absence of FK506 effects in females may reflect compensatory or hormone-dependent mechanisms rather than reduced drug efficacy, reinforcing the importance of considering sex as a biological variable in addiction research.

We further evaluated whether FK506 would be able to alter another abnormal behavior induced by cocaine. For this, we used the CPP paradigm, a common model used to study substance use disorders. While the behavior sensitization is based on the enhancement of drug-induced locomotor activity following repeated passive exposure to a fixed drug dose, the CPP evaluates associative learning and the rewarding properties of the drug (Kuhn et al., 2019). Therefore, these models evaluate complementary effects of cocaine. Interestingly, although FK506 attenuated locomotor sensitization in males, it failed to alter cocaine-induced CPP in both sexes. These results indicate that FK506 preferentially influences neuroadaptations associated with motor sensitization, but not the mechanisms underlying cocaine reward and associative memory. CPP depends on the recruitment and reactivation of sparse hippocampal neuronal ensembles within specific subregions, rather than on global molecular changes across the hippocampus (Trouche et al., 2016). Consequently, molecular alterations detected in bulk tissue may fail to capture synaptic or circuit-level adaptations occurring selectively within the neuronal populations that encode reward–context associations. In this context, FK506 induced changes in cytokines (Liu et al., 2017; Kocanci and Goksu, 2024) and GDNF levels are likely to modulate hippocampal excitability, inflammatory tone, or neurotrophic support without necessarily interfering with the synaptic mechanisms required for memory encoding or retrieval underlying CPP (Ghitza et al., 2010). Moreover, calcineurin signaling has been more strongly implicated in forms of plasticity associated with stress responses, neuroimmune modulation, and behavioral sensitization than with the consolidation or expression of associative memories (Baumgärtel and Mansuy, 2012). Thus, inhibition of calcineurin may preferentially influence motivational or sensitization-related adaptations while leaving hippocampal-dependent reward learning largely intact. Despite the behavioral effects observed, most molecular and structural outcomes measured in this study remained unaffected by FK506. Repeated cocaine exposure reduced dendritic spine density in both the striatum and hippocampus, and FK506 did not prevent these cocaine-induced structural alterations. This effect induced by cocaine may seem contradictory with the literature. Classic studies in rats have shown that long-term cocaine administration (approximately 4 weeks) typically increases dendritic spine density in brain regions (Robinson and Kolb, 1999; Norrholm et al., 2003; Li et al., 2004; Lee et al., 2006). A work in mice also demonstrated increased hippocampal spine density, although the authors did not describe the subregion evaluated (Ka et al., 2016). However, not all findings are in accordance with these previous studies. Shen et al. (2009) reported no significant spine alterations after chronic cocaine exposure in rats. Also, acute cocaine administration has been shown to decrease dendritic spines in the prelimbic prefrontal cortex (Sequeira et al., 2023). In our studies, we used C57Bl/6 mice that received a total of 5 cocaine injections. Taken together, these discrepancies suggest methodological differences across studies, such as species, brain region, treatment duration, doses and timing of tissue collection, may explain why cocaine produced spine reductions under our experimental conditions. Notably, FK506 alone also reduced dendritic spine density in the hippocampus, suggesting a region-specific effect that appears to be independent of cocaine exposure. To our knowledge, no previous study has reported hippocampal spine reduction induced by this drug, which suggests that this should be investigated in future works.

In addition to the dendritic spines, transcriptional levels of PSD-95, CREB, Arc, and FosB were also unaltered across all groups. The lack of detectable gene expression changes may reflect the specific timing of tissue collection, considering that some transcriptional regulation induced by cocaine may be transient. It is also possible that the attenuation of behavioral sensitization by FK506 relies on mechanisms that do not involve changes in these synaptic plasticity markers, but in modulation of intracellular signaling pathways or neurotransmitter dynamics.

Recent studies have highlighted the contribution of neuroinflammation and CNS immune modulators to psychostimulant-induced behavioral alterations. In particular, microglia mediate the accumulation of calcium-permeable AMPA receptors in the nucleus accumbens, driving hyperlocomotion during cocaine withdrawal (Reverte et al., 2024). These findings indicate that immune cells actively contribute to the remodeling of excitatory synapses and the expression of locomotor sensitization. Recently our group demonstrated that inhibition of the colony-stimulating factor 1 receptor (CSF1R), which is essential for microglial viability, alters both behavioral and molecular responses to cocaine, highlighting a role for microglia in cocaine-induced neuroadaptations (da Silva et al., 2021). In regards to neuroinflammatory mediators, chronic cocaine exposure activates microglia and alters cytokine profiles, typically increasing proinflammatory mediators such as TNF and IL-1β while decreasing anti-inflammatory cytokines like IL-10 (Hutchinson et al., 2012; Northcutt et al., 2015). In this study, FK506 decreased hippocampal TNF and IL-10 levels, consistent with the immunomodulatory effects expected from calcineurin inhibition. Cytokine regulation is region- and context-dependent. The increase in IL-10 in the striatum, but not in the hippocampus, following cocaine exposure may reflect region-specific microenvironmental influences or distinct temporal dynamics of cytokine release. IL-10 can exert neuroprotective or permissive effects depending on the phase and intensity of neuroimmune activation (Levandowski et al., 2016; Lacagnina et al., 2017). Likewise, the FK506-induced reduction of TNF aligns with evidence that proinflammatory cytokines modulate dopaminergic signaling and synaptic remodeling in addiction (Lewitus et al., 2016; Gonçalves et al., 2017).

Although we did not directly assess the cellular mechanisms underlying FK506 treatment, this compound likely acts through broad inhibition of calcineurin in multiple cell types, including neurons, astrocytes, and microglia. While calcineurin is well known for its neuronal functions, accumulating evidence indicates that it also plays critical roles in glial cells, although its involvement in SUD remains poorly characterized. In reactive astrocytes, calcineurin signaling regulates neuroinflammatory responses by modulating the balance between pro-inflammatory and anti-inflammatory pathways and by coordinating morphological and inflammatory changes (Fernandez et al., 2007; Furman and Norris, 2014). Moreover, astrocytic calcineurin has been implicated in synaptic remodeling, neuronal hyperexcitability, and Ca^2+^ homeostasis under pathological conditions (Furman and Norris, 2014; Furman et al., 2016; Sompol et al., 2017). In microglia, calcineurin/NFAT signaling controls microgliosis and the production of inflammatory mediators (Nagamoto-Combs and Combs, 2010; Rojanathammanee et al., 2015; Jiang et al., 2022). Considering that neuroinflammation, synaptic plasticity, neuronal excitability, and Ca^2+^ homeostasis are key processes involved in SUD, it is plausible that glial calcineurin signaling contributes to the effects observed here, warranting further investigation.

Finally, we observed that cocaine increased GDNF in the hippocampus, an effect that was reversed by FK506, albeit BDNF and NGF were not altered. GDNF is widely recognized for its role in supporting dopaminergic neuron survival, promoting synaptic plasticity, and preserving dendritic architecture (Ron and Janak, 2005; Jain et al., 2006; Ghitza et al., 2010). Interestingly, despite the increase in hippocampal GDNF, cocaine exposure in the present study was associated with a reduction in dendritic spine density in the same region. This apparent discrepancy suggests that cocaine-induced upregulation of GDNF may represent a compensatory or homeostatic response rather than a sufficient protective mechanism. Under conditions of repeated cocaine exposure, neurotrophic signaling may be uncoupled from its canonical structural effects due to concurrent neuroinflammatory signaling, excitotoxic stress, or alterations in downstream effectors of GDNF signaling. Thus, increased GDNF levels may reflect an attempt to counteract synaptic destabilization rather than an indicator of preserved synaptic integrity. The reversal of GDNF elevation by FK506 further suggests that calcineurin-dependent pathways contribute to cocaine-induced neurotrophic adaptations. However, the persistence of spine loss despite GDNF normalization indicates that FK506-sensitive signaling is not sufficient to rescue cocaine-induced structural plasticity.

## 5 Conclusion

Taken together, these findings indicate that FK506 exerts selective effects on cocaine-induced neuroadaptations. While the drug attenuated locomotor sensitization in males and modulated hippocampal inflammatory and neurotrophic responses, it did not influence cocaine reward, dendritic spine density altered by cocaine, or the expression of classic plasticity-related genes. The dissociation between behavioral and molecular outcomes highlights the complexity of cocaine-induced adaptations and suggests that FK506 may act on specific neuroimmune mechanisms rather than synaptic remodeling pathways. Further studies are necessary to clarify the sex-dependent effects and the neurobiological targets influenced by this immunosuppressant.

## Data Availability

The original contributions presented in the study are included in the article/Supplementary Material, further inquiries can be directed to the corresponding author.
