# Supplementary material for: Immunosuppressant treatment reduces cocaine-induced behavioral sensitization in mice
Source: Front Pharmacol. 2026 Feb 10;17:1757023. doi: 10.3389/fphar.2026.1757023 (PMC12929953; doi:10.3389/fphar.2026.1757023)
Supplement: Supplementary file 1 [file DataSheet1.pdf]

Supplementary Material

1 Supplementary Figures and Tables

1.1 Supplementary Figures

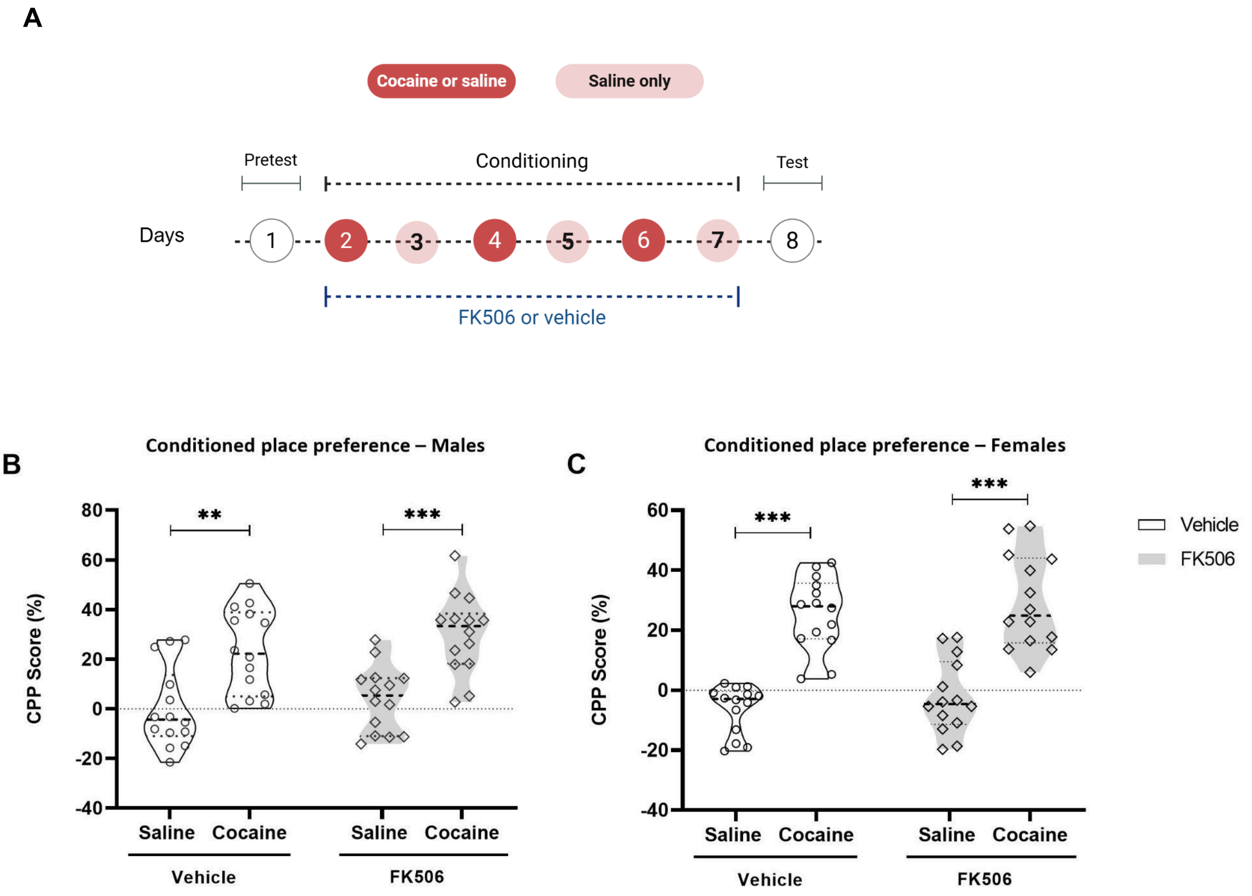

Supplementary Figure 1. Conditioned place preference in male and female mice treated with FK506. (A) Experimental timeline illustrating the pretest (day 1) and the conditioning period with daily treatments (days 2-7), followed by the test (day 8). Analysis of preference scores in (B) males (FK506 + cocaine, n=23; vehicle + cocaine, n=23; FK506 + saline, n=22; vehicle + saline, n=21) and (C) females (FK506 + cocaine, n=18; vehicle + cocaine, n=17; FK506 + saline, n=18; vehicle + saline, n=15). Bars represent the mean ± SEM of the percentage of time spent in the drug-paired compartment. Data were analyzed by two-way ANOVA followed by Tukey's post hoc test. ** p<0,01; ***p<0.001..

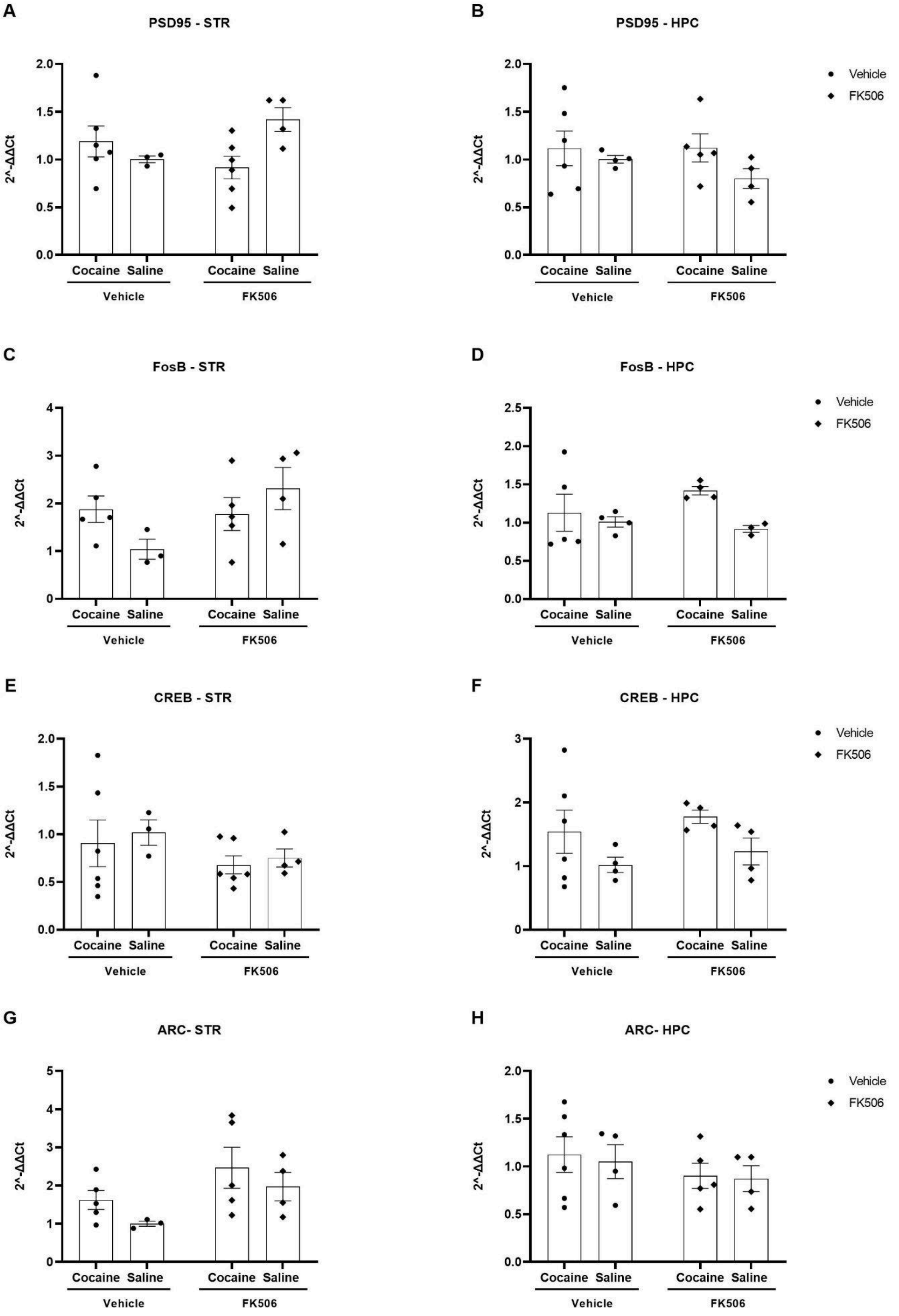

Supplementary Figure 2. Expression of synaptic plasticity markers in the striatum (STR) and hippocampus (HPC) following FK506 treatment. Quantitative PCR analysis was performed for PSD-95 (A-B for striatum and hippocampus, respectively), FosB (C-D for striatum and hippocampus, respectively), CREB (E-F for striatum and hippocampus, respectively), and Arc (G-H for striatum and hippocampus, respectively). Groups included cocaine + FK506 (n=6), cocaine + vehicle (n=6), FK506 + saline (n=4), and saline + vehicle (n=3). Data represent mean ± SEM. Two-way ANOVA followed by Tukey's post hoc test showed no significant differences between groups.
